# Natural history of multiple myeloma with de novo del(17p)

**DOI:** 10.1038/s41408-019-0191-y

**Published:** 2019-03-07

**Authors:** Arjun Lakshman, Utkarsh Painuly, S. Vincent Rajkumar, Rhett P. Ketterling, Prashant Kapoor, Patricia T. Greipp, Morie A. Gertz, Francis K. Buadi, Martha Q. Lacy, David Dingli, Angela Dispenzieri, Amie L. Fonder, Suzanne R. Hayman, Miriam A. Hobbs, Wilson I. Gonsalves, Yi Lisa Hwa, Nelson Leung, Ronald S. Go, Yi Lin, Taxiarchis V. Kourelis, Rahma Warsame, John A. Lust, Stephen J. Russell, Steven R. Zeldenrust, Robert A. Kyle, Shaji K. Kumar

**Affiliations:** 1 Division of Hematology, Mayo Clinic, Rochester, MN USA; 2 4th Department of Internal Medicine - Hematology, University Hospital Hradec Kralove and Charles University in Prague, Faculty of Medicine in Hradec Kralove, Czech Republic, Hradec Kralove, Czech Republic; 3 Department of Laboratory Medicine and Pathology, Mayo Clinic, Rochester, MN USA

## Abstract

We compared the outcomes of 310 patients with newly diagnosed multiple myeloma with del(17p) detected by FISH to patients with high-risk translocations (HRT) (*n* = 79) and standard-risk (SR) cytogenetics (*n* = 541). The median progression-free survival (PFS) following initial therapy for the three groups was 21.1, 22, and 30.1 months, respectively (*P* = 0.437- del(17p) vs. HRT); the median overall survival (OS) was 47.3, 79.1, and 109.8 months, respectively, (*P* = 0.007- del(17p) vs. HRT). PFS and OS for patients with relative loss of 17p (*n* = 21) were comparable to other patients with del(17p). The PFS was similar between the del(17p) and HRT groups when stratified for age, ISS stage or treatment. The OS of del(17p) and HRT groups were similar in presence of advanced age, ISS III stage or if patients did not receive a proteasome-inhibitor containing induction. ISS III stage, high LDH and HRT, but not the percentage of cells with del(17p) predicted shorter OS in patients with del(17p). The median OS for low (ISS I, normal LDH and no HRT), intermediate (neither low nor high-risk) and high-risk (ISS III and either elevated LDH or coexistent HRT) groups among del(17p) patients were 96.2, 45.4, and 22.8 months, respectively, allowing further risk stratification.

## Introduction

Multiple myeloma (MM) is the second most common hematological malignancy and was expected to cause over 12,000 deaths in the United States in 2018^1^. Translational research with introduction of proteasome inhibitors (PIs) and immunomodulatory drugs (IMiDs) and use of high-dose therapy with autologous stem cell transplant (ASCT) has consistently improved the outcomes in patients with MM over the past 2 decades^2,3^. MM is clinically and biologically heterogeneous and outcomes are influenced by a variety of factors including patient characteristics, tumor load, and disease biology including the genomic abnormalities in malignant plasma cells (PCs)^4–6^. Recurrent cytogenetic abnormalities detected by interphase fluorescent in situ hybridization (FISH) on malignant PCs give key prognostic information^7–9^. Deletion of short arm of chromosome 17 (17p13.1), is one of the adverse cytogenetic markers at diagnosis^10^.

FISH detects del(17p) in 5–20% patients with newly diagnosed MM (NDMM) depending on the cut-off used^8,9,11–20^. Del(17p) occurs more frequently at relapse and in aggressive disease including PC leukemia and central nervous system relapse^21–23^. Hemizygous del(17p) has consistently been associated with shorter progression free survival (PFS) and overall survival (OS) in patients with MM^7–9,11,14,15,19,20,24–31^. Loss of the *TP53* gene located at 17p13.1 locus is thought to be at least partly responsible for the adverse outcomes^32^. *TP53* is a critical regulator of cell cycle and apoptosis^33^. *TP53* mutations are rare in NDMM, seen in up to 3% patients, and appear to occur at a higher frequency in patients with del(17p)^34–38^. Patients with biallelic inactivation of *TP53* have an aggressive clinical course and poor prognosis^15,39,40^. Being a secondary cytogenetic abnormality, del(17p) coexists with other FISH abnormalities, which in turn may influence the outcomes in these patients^18,27,29–31,41^. Improving the outcomes of these patients will require a concerted effort with well-designed clinical trials within this patient group. In order to do that, an accurate assessment of the natural history of these patients is essential. In this study, we report the long-term outcomes of a large cohort of MM patients with del(17p) treated at our center. Further, we compare them with patients with high-risk chromosomal translocations and standard risk FISH and evaluate the specific factors at diagnosis, which influence outcomes in patients with del(17p).

## Patients and methods

### Patients

We reviewed the Dysproteinemia database at Mayo Clinic, Rochester and electronic medical records, to identify patients with MM who underwent FISH testing between 2004 and August 2016 and demonstrated del(17p) at diagnosis or within 6 months of the diagnosis of MM. De novo del(17p) was defined as del(17p13.1), which includes the p53 gene region, and/or monosomy for chromosome 17. Relative loss of 17p was defined as del(17p) in presence of trisomy or tetrasomy involving chromosome 17. We excluded all patients who had MM with an amyloid related systemic syndrome (*n* = 4) or PC leukemia before the index FISH (*n* = 31), or for whom details about initial therapy were not available (*n* = 11)^42^. Three hundred and ten (310) patients satisfied the inclusion criteria. For each patient with del(17p), we identified two patients with MM matched for age and time period of diagnosis, who did not have del(17p) by FISH within 6 months from diagnosis and satisfied the other inclusion criteria. We subdivided the control group (*n* = 620) into a high-risk translocation (HRT) group [with t(4;14), t(14;16) or t(14;20)] (*n* = 79) and a standard-risk (SR) group (*n* = 541) for comparing the outcomes. The Mayo Clinic Institutional Review Board approved the study. The study was conducted in accordance with the Declaration of Helsinki and the Health Insurance Portability and Accountability Act guidelines of 1996.

We collected data regarding demographic characteristics, pre-treatment laboratory parameters, treatment administered, best response to induction, progression, and survival status at final data cut-off by retrospective chart review. The data cut-off date was 31 January 2018. In all patients, diagnosis of MM was made based on the standard criteria, which were in use during the defined period^42,43^. We used the international staging system (ISS) to risk stratify patients where serum beta-2-microglobulin and albumin levels were available before starting treatment^4^. High PC proliferative rate was defined by a PC labelling index of ≥1.5% or a monotypic PC- S-phase fraction of ≥3% in the flow cytometric PC proliferation study^44^. We assessed the best response to induction using the International Myeloma Working Group (IMWG) consensus response criteria^45^. Clinical benefit rate (CBR) was defined as the proportion of patients who obtained at least a minor response as the best response to induction. We defined ‘early SCT’ as SCT done within 12 months of starting treatment for MM.

### Outcome measures

Our primary outcome was OS, defined as duration from diagnosis of MM to death due to any cause, patients being censored if they were alive at the last follow-up^46^. Secondary outcome measures included best response to induction therapy and PFS. We defined overall response rate (ORR) as the proportion of patients attaining a partial response (PR) or better following induction. We defined PFS as the duration from the initiation of treatment to first progression or death due to any cause and we censored patients who were alive without progression at their last follow-up^46^.

### FISH

Bone marrow aspirate samples enriched for mononuclear cells by the Ficoll method were used for preparing cytospin slides. Cytoplasmic immunoglobulin staining was used to identify plasma cells and the FISH analysis was performed as described previously from our institution using the following probes: 3cen (D3Z1), 7cen (D7Z1), 9cen (D9Z1), 15cen (D15Z4), 11q13 (CCND1-XT), 13q14 (RB1), 13q34 (LAMP1), 17p13.1 (p53), 17cen (D17Z1), 14q32 (IGH-XT), 14q32 (3′IGH,5′IGH), 4p16.3 (FGFR3), 16q23 (c-MAF), 6p21 (CCND3), 20q12 (MAFB), 1p (p73), and (1q22)^8^. The cut-points for a positive test were 7 and 9% for deletion 17p13.1 and monosomy 17, respectively. Hyperdiploidy was defined as presence of trisomy/tetrasomy of ≥2 odd-numbered chromosomes.

### Statistical analysis

We summarized categorical variables as proportions and continuous variables as medians (range). We used Fisher’s exact test to compare categorical variables and the non-parametric Mann–Whitney U and Kruskall–Wallis tests as appropriate to compare continuous variables between groups. We estimated PFS and OS using the Kaplan–Meier method and used the log-rank test to compare them between groups. We used the Cox proportional hazards model to identify baseline factors affecting PFS and OS. A two-tailed *p*-value <0.05 was considered significant for all statistical tests. We used JMP® Pro 12.0 software package (SAS Institute Inc., Cary, NC, USA) for all statistical analyses.

## Results

A comparison of the baseline demographic and laboratory characteristics across the patient groups is given in Table 1 and the other cytogenetic abnormalities detected are given in Table 2. A higher proportion of patients with del(17p) had a higher PC proliferative rate and elevated lactate dehydrogenase (LDH) at diagnosis. HRTs were more likely to coexist in the del(17p) group (24.2%) when compared to the control group (12.7%) (*P* < 0.001). Among patients for whom testing for del(1p) and del(1q) were available, they were detected at a similar frequency in the del(17p) (31.9%) and the control group (31.1%) (*P* = 0.908). Overall, any high-risk abnormality other than del(17p) occurred in 31.6% patients with del(17p) and 17.7% patients in the control group (*P* < 0.001). Out of 310 patients in the del(17p) group, 246 (79.4%) patients had del(17p13.1) and 41 (13.2%) patients had monosomy 17. Two patients (0.6%) had concurrent del(17p13.1) and monosomy 17. Relative loss of 17p was present in 21 (6.8%) patients; 3 of these patients had tetraploidy while others had trisomy/tetrasomy. The median follow-up for all the patients was 63.5 months (95% CI, 58.3–67.5); 54.5 (95% CI, 49.8–66.9), and 65.7 (95% CI, 59.2–71.3) months for the del(17p) and control groups, respectively. At data cut-off, 169 (54.5%), 32 (40.5%) and 174 (32.2%) patients, respectively in del(17p), HRT and SR groups had died.

**Table 1:** Demographic and laboratory characteristics of the study population at diagnosis (*n* = 930)

Characteristic	Del(17p) (*n* = 310)	All control patients (*n* = 620)	High-risk translocation (*n* = 79)	Standard-risk (*n* = 541)	*P* ^a^
Age at diagnosis, median (range)	64.1 (33.8–90.9)	64.2 (35.2–91.0)	60.4 (37.1–81.2)	64.8 (35.2–91.0)	0.787; 0.060
Age ≥65 years, *n* (%)	147 (47.4)	296 (47.7)	29 (36.7)	267 (49.3)	0.945; 0.108
Female gender, *n* (%)	122 (39.3)	242 (39.0)	41 (51.9)	201 (37.1)	0.924; **0.044**
*Time period of diagnosis of MM*					
2004–2008	34 (11.0)	71 (11.4)	11 (13.9)	60 (11.1)	0.536; 0.950
2008–2012	139 (44.8)	282 (45.5)	29 (36.7)	253 (46.8)	
2013–2016	137 (44.2)	267 (43.1)	39 (49.4)	228 (42.1)	
Hemoglobin, g/L, median (range), (*n* = 907)	10.7(4.7–16.8)	11.2 (5.8–16)	10.6 (5.8–14.7)	11.2 (5.9–16.0)	**0.015**; **0.005**
Calcium, mg/dL, median (range), (*n* = 849)	9.7 (7.7–16.8)	9.6 (7.1–17.1)	9.4 (7.7–16.6)	9.6 (7.1–17.1)	0.089; **0.268**
Creatinine >2 mg/dL, n(%), (*n* = 883)	52 (18.4)	82 (13.8)	14 (18.7)	68 (12.9)	0.071; 0.121
Bone disease at diagnosis, *n* (%)	243 (78.4)	474 (76.4)	48 (60.8)	426 (78.4)	0.562; **0.003**
Lytic lesions, *n* (%)	205 (66.1)	419 (67.6)	45 (57.0)	374 (69.1)	0.658; 0.092
Pathological fractures, *n* (%)	50 (16.1)	104 (16.8)	6 (7.6)	98 (18.1)	0.852; 0.050
Vertebral compression fractures, *n* (%)	108 (34.8)	403 (65.0)	18 (22.8)	199 (36.8)	1.0; **0.048**
Bone marrow PC percentage, median (range), (*n* = 920)	50 (2–100)	50 (2–100)	50 (10–100)	46 (2–100)	**0.033**; **0.013**
High PC proliferative rate, n (%) (*n* = 504)	42 (30.0)	59 (16.1)	7 (16.3)	52 (16.1)	**<0.001**; **0.003**
M-protein level, g/dL, median (range), (*n* = 876)	2.3 (0–8.2)	2.6 (0–10)	3.7 (0–9)	2.5 (0–10)	0.154; <**0.001**
M-protein isotype, *n* (%)					
IgG	176 (56.8)	367 (59.2)	46 (58.2)	321 (59.3)	
IgA	64 (20.7)	136 (22.0)	27 (34.2)	109 (20.1)	0.605; **0.031**
Light chain only	60 (19.3)	100 (16.1)	5 (6.3)	95 (17.6)	
Others	10 (3.2)	17 (2.7)	1 (1.3)	16 (3.0)	
Difference between involved and uninvolved free light chain, mg/dL, median (range), (*n* = 780)	61.3 (0–2589.0)	46.2 (0–6620)	40.53 (0.4–1999.7)	46.9 (0–6620)	0.126; 0.212
Risk stratification, *n* (%)					
ISS I/II (*n* = 541)	154 (62.3)	387 (69.2)	44 (63.8)	343 (70.0)	0.061; 0.092
ISS III (*n* = 265)	93 (36.7)	172 (30.8)	25 (36.2)	147 (30.0)	
Elevated LDH, (*n* = 671)	49 (23.8)	66 (14.2)	7 (13.0)	59 (14.4)	**0.004**; **0.012**

ISS indicates international staging system

*MM* multiple myeloma, *LDH* lactate dehydrogenase, and *PC* plasma cell

^a^*p*-value for Fischer’s exact test or the non-parametric Mann-Whitney U or Kruskall Wallis tests as appropriate. The first value represents comparison between the de novo del(17p) and all controls and the second value represents comparison across de novo del(17p), high-risk translocation and standard-risk groups

The values given in bold represent *P*-values <0.05, which are considered statistically significant

**Table 2:** Cytogenetic profiles of patients based on interphase fluorescent in situ hybridization (*n* = 930)

Cytogenetic abnormality	De novo del(17p) (*n* = 310)	All control patients (*n* = 620)	High-risk translocation (*n* = 79)	Standard-risk (*n* = 541)	*P* ^a^
t(4;14)	48 (15.5)	49 (7.9)	49 (62.0)	–	<0.001
t(6;14)^b^	4 (1.7)	7 (1.5)	–	7 (1.7)	1.000
t(11;14)	45 (14.5)	131 (21.1)	–	131 (24.2)	0.016
t(14;16)	24 (7.7)	22 (3.2)	22 (27.8)	–	0.009
t(14;20)^b^	3 (1.2)	9 (1.9)	9 (13.8)	–	0.760
Unspecified immunoglobulin heavy chain (IgH) rearrangement/IgH variable region deletion	19 (6.1)	56 (9.0)	–	56 (10.3)	0.159
Any trisomy or tetrasomy	154 (49.7)	345 (55.6)	31 (39.2)	314 (58.0)	0.002; 0.094
Hyperdiploidy	112 (36.1)	276 (44.5)	16 (20.2)	260 (48.1)	0.016; <0.001
Del (13q) and/or monosomy 13	200 (64.5)	257 (41.4)	64 (81.0)	193 (35.7)	<0.001; <0.001
Del (13q)	39 (12.6)	51 (8.2)	11 (13.9)	40 (7.4)	0.045; 0.017
Monosomy 13	163 (52.6)	213 (34.3)	57 (72.5)	156 (28.8)	<0.001; <0.001
1q22 gain^c^	21 (29.2)	38 (31.1)	11 (68.7)	27 (25.5)	0.872; 0.003
Del (1p31)^c^	4 (5.6)	1 (0.8)	1 (6.2)	0 (0)	0.064; 0.021

^a^*p*-value for Fischer’s exact test. Comparison between del(17p) and all controls when only one value is present and the second value when present represents comparison across del(17p), high-risk translocation and standard-risk groups

^b^Calculation is limited to patients who had FISH after May 2009 (*n* = 716) when probes for these abnormalities were introduced

^c^Calculation limited to patients who had FISH after August 2014 (*n* = 194) when probe for 1q gain and del (1p) were introduced

### Induction therapy and response to induction

**Fig. 1:**
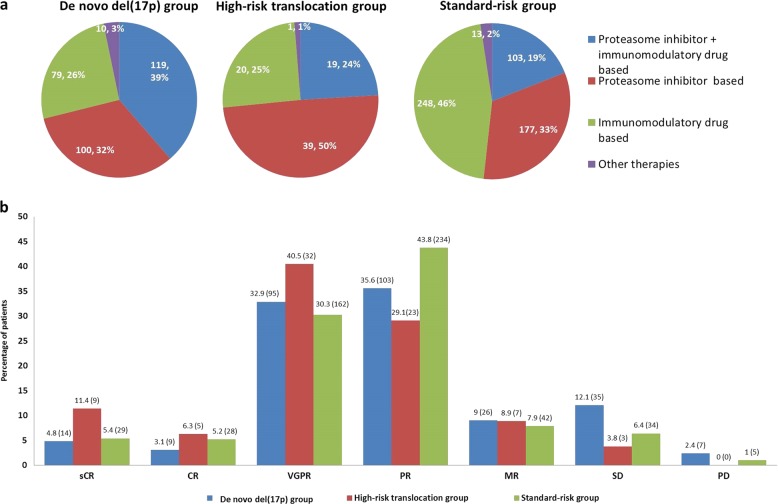
Induction therapy and response to induction in the three groups. **a** Proportion of patients receiving proteasome inhibitor + immunomodulatory drug based, proteasome inhibitor based, immunomodulator based, and other induction therapies in patients with (i) de novo del(17p), (ii) high-risk translocation and (iii) standard-risk FISH. **b** Best response to induction therapy in patients with de novo del(17p), high-risk translocation and standard-risk FISH, expressed as percentage within each category. The numbers in parentheses indicate the absolute number of patients. CR complete response, MR minimal response, PR partial response, PD progressive disease, sCR stringent CR, VGPR very good partial response, and SD Stable disease

The major classes of induction therapy received by patients in the del(17p), HRT, and SR groups are shown in Fig. 1a. Patients with del(17p) and HRT were more likely to receive a PI-containing regimen (71.1 and 73.4%, respectively) when compared to SR patients (51.8%) (*P* < 0.001). Best response to induction was evaluable in 289 (93.2%), 79 (100%) and 534 (98.7%) patients, respectively, in the three groups. The best responses obtained during induction in these patients are shown in Fig. 1b. ORR was lower in patients with del(17p) (76.5%) compared to those patients with HRT (87.3%) or SR disease (84.8%) (*P* = 0.006). Details are given in supplementary material. Among patients who received PI + IMiD- based induction, the ORRs were 85.4, 94.7, and 97.1% in the three groups (*P* = 0.009), while VGPR or better rates in the three groups were 52.7, 78.9, and 63.7%, respectively (*P* = 0.054). Similarly, in patients who received a PI-containing regimen, the ORRs were 78.7, 93.1, and 84.4%, respectively, in the three groups (*P* = 0.026) and VGPR or better rates were 28.2, 23.8, and 32.2%, respectively (*P* = 0.615).

### Survival outcomes

**Fig. 2:**
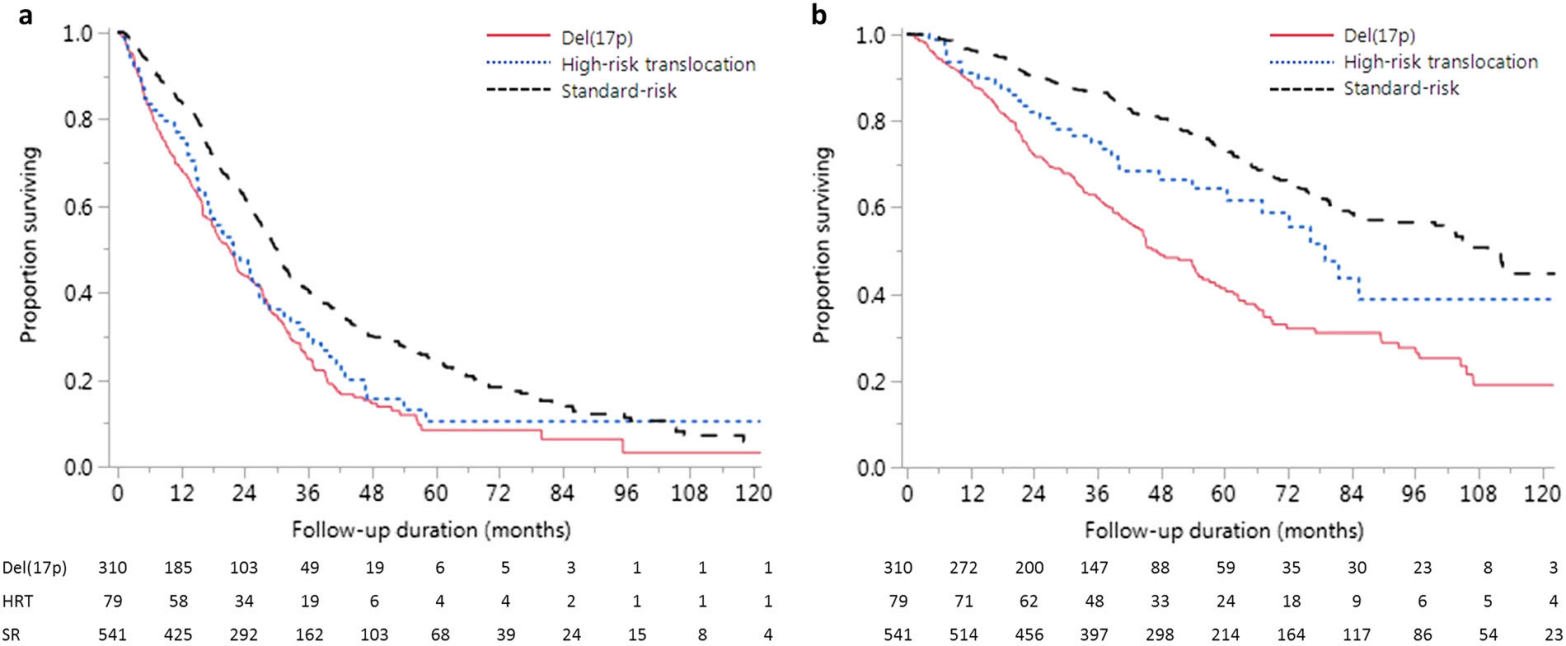
Survival outcomes in the three groups. Kaplan–Meier survival curves showing comparison of **a** progression-free survival (PFS), **b** overall survival (OS) between patients with del(17p), high-risk translocation (HRT) and standard-risk (SR) FISH. For PFS, *P* = 0.437 for del(17p) vs. HRT and *P* < 0.001 for del(17p) vs. SR; and for OS, *P* = 0.007 for del(17p) vs. HRT and *P* < 0.001 for del(17p) vs. SR

The estimated median PFS for del(17p), HRT and SR groups were 21.1 months (95% CI, 17.8–23.9), 22.0 months (95% CI, 16.7–26.8) and 30.1 months (95% CI, 27.5–31.5) respectively (*P* = 0.437 for del(17p) vs. HRT and *P* < 0.001 for del(17p) vs. SR) (Fig. 2a). The estimated median OS for the three groups were 47.3 months (95% CI, 42.7–55.9), 79.1 months (95% CI, 60.5-not reached[NR]), and 109.8 months (95% CI, 99.9–125.6), respectively, (*P* = 0.007 for del(17p) vs. HRT and *P* < 0.001 for del(17p) vs. SR) (Fig. 2b). The median PFS for patients with relative loss of 17p was 22.1 months (95% CI, 8.7–51.8) and was comparable to 21.2 months (95% CI, 17.8–25.0) seen in patients with del17p or monosomy 17 (*P* = 0.485). Similarly, the median OS for the two groups were comparable: 48.7 months (95% CI, 32.1-NR) vs. 47.3 months (95% CI, 41.6–55.9) (*P* = 0.603), justifying their inclusion in the del(17p) group (Supplementary material).

**Fig. 3:**
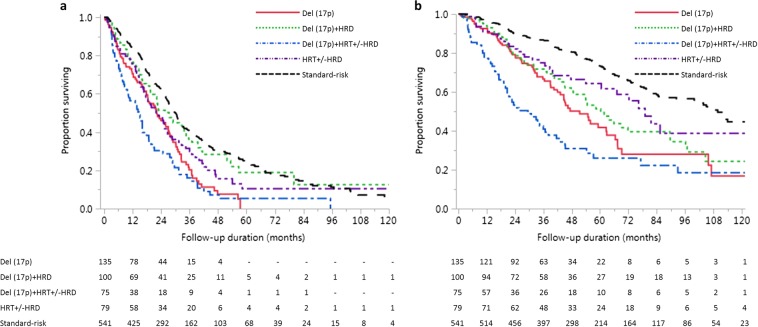
Survival outcomes in patients with combinations of cytogenetic abnormalities. Kaplan–Meier curves showing comparison of **a** Progression-free survival (PFS) and **b** overall survival (OS) between patients with del(17p) alone (*n* = 135), del(17p) with hyperdiploidy (HRD) (*n* = 100), del(17p) with high-risk translocation (HRT) (*n* = 75), HRT (*n* = 79) and standard-risk patients (*n* = 541). For PFS and OS, *P* < 0.001 for log-rank test

To elucidate the impact of combinations of FISH abnormalities, we divided the entire patient cohort (cases and controls) into the following groups: cases were divided into del(17p) alone (*n* = 135), del(17p) with hyperdiploidy (*n* = 100), del(17p) with HRT (irrespective of presence of hyperdiploidy) (*n* = 75), and controls were divided into HRT (irrespective of presence of hyperdiploidy) (*n* = 79) and SR patients (*n* = 541). The median PFS in the above five groups were 22.4 months (95% CI, 17.8–27.0), 27.3 months (95% CI, 19.6–34.5), 14.7 months (95% CI, 9.8–17.9), 22.0 months (95% CI, 16.7–26.8), and 30.1 months (95% CI, 27.5–31.5), respectively, (*P* < 0.001). The median OS in the above five groups were 51.4 months (95% CI, 42.1–62.8), 60.3 months (95% CI, 47.8–89.6), 29.5 months (95% CI, 20.0–38.1), 79.1 months (95% CI, 60.5-not reached), and 109.8 months (95% CI, 99.9–125.6), respectively, (*P* < 0.001) **(**Fig. 3**)**. Presence of hyperdiploidy was associated with longer PFS (*P* = 0.007) and only a trend toward longer OS (*P* = 0.272) in del(17p) patients. Coexistent HRT worsened the OS (*P* = 0.004).

### Use of SCT

Among patients with del(17p), HRT, and SR disease, 173 (55.8%), 55 (69.6%), and 325 (60.1%) received SCT at any time during their disease course (*P* = 0.071). Within the three groups, 187 (60.3%), 61 (77.2%), and 368 (68.0%) patients, respectively, were transplant-eligible and 159 (85.0%), 43 (70.5%), and 264 (71.7%) transplant-eligible patients in the three groups underwent early SCT. Among transplant-eligible patients, an early SCT was not associated with longer median OS in all the three groups: 54.9 months (95% CI, 45.3–66.5) vs. 77.2 months (95% CI, 44.6-NR) in del(17p) group (*P* = 0.589); 85.4 months (95% CI, 72.3-NR) vs. NR (95% CI, 38.7-NR) in HRT group (*P* = 0.794); and 130.6 months (95% CI, 10.6.1-NR) vs. 125.6 months (95% CI, 105.6-NR) in SR group (*P* = 0.549).

### Sub-group analysis for survival outcomes

We stratified patients according to age (using 65 years as cut-off), ISS stage, LDH, and PC proliferative rate at diagnosis and the type of induction therapy. The results are shown in Table 3. There was no difference in PFS between patients with del(17p) and HRT across all the subgroups analyzed. The OS was shorter in del(17p) group compared to the HRT group in patients with age <65 years, ISS I/II stage and those patients who received PI-containing induction regimen or early SCT. However, the difference was abolished and both groups had similar OS in presence of adverse factors, such as advanced age, ISS III stage or when they received non-PI containing induction regimens or delayed or no SCT. This loss of difference in OS was primarily due to a marked reduction in OS in the HRT group in presence of additional risk factors as shown in Table 3. For example, in the del(17p) group, ISS I/II and ISS III stages were associated with median OS of 58.3 and 33.3 months, respectively, while in the HRT group, the OS decreased from 81.6 months in ISS I/II stages to 38.7 months in ISS III stage. Similarly, a non-PI-containing induction was associated with reduction of median OS from 54.3 months in the del(17p) group to 45.2 months, while it decreased from not reached to 67.1 months in the HRT group. Across all subgroups, the SR group showed consistently better outcomes when compared to the del(17p) group.

**Table 3:** Sub-group analysis for survival outcomes in patients based on prognostic factors and therapy

Survival outcomes and subgroups	De novo del(17p)	High-risk translocation	Standard-risk	*P* ^a^
*Progression free survival, (months)*				
Age <65 years (*n* = 487)	21.0 (16.8–27.0)	25.4 (17.5–32.6)	32.3 (28.7–37.7)	0.176; <**0.001**
Age ≥65 years (*n* = 443)	21.3 (16.0–25.8)	16.6 (11.2–25.5)	27.4 (25.3–30.6)	0.520; **0.002**
ISS I/II (*n* = 541)	27.0 (21.1–30.3)	24.6 (17.1–31.5)	30.9 (29.1–34.8)	0.699; **0.002**
ISS III (*n* = 265)	14.3 (9.6–16.9)	16.7 (5.1–34.4)	24.8 (19.7–28.5)	0.212; <**0.001**
PI-containing induction (*n* = 557)	22.6 (18.4–27.5)	25.0 (18.1–32.6)	29.5 (26.3–31.8)	0.336; <**0.001**
Others (*n* = 371)	16.1 (13.8–22.0)	13.3 (5.1–25.5)	30.6 (27.1–33.8)	0.840; <**0.001**
Normal LDH (*n* = 556)	22.5 (18.4–28.2)	18.4 (15.1–25.4)	30.8 (28.0–33.1)	0.241; <**0.001**
High LDH (*n* = 115)	16.1 (8.3–17.9)	6.7 (1.5–21.9)	27.1 (18.3–32.3)	0.245; <**0.001**
Low PC proliferative rate (*n* = 405)	22.3 (17.8–28.8)	16.6 (13.3–22.0)	29.8 (26.3–32.1)	0.106; **0.044**
High PC proliferative rate (*n* = 101)	10.4 (5.1–18.6)	6.7 (2.3–17.1)	25.0 (17.9–31.3)	0.264; <**0.001**
*Overall survival, (months)*				
Age < 65 years (*n* = 487)	55.2 (42.0–67.4)	81.6 (60.5-NR)	130.6 (112.6-NR)	**0.030**; <**0.001**
Age ≥ 65 years (*n* = 443)	44.7 (37.5–54.6)	67.1 (25.2-NR)	78.6 (70.4–103.6)	0.201; <**0.001**
ISS I/II (*n* = 541)	58.3 (45.3–71.8)	81.6 (60.5-NR)	112.3 (103.6-NR)	**0.039**; <**0.001**
ISS III (*n* = 265)	33.3 (23.2–44.7)	38.7 (21.1-NR)	64.7 (59.6–124.4)	0.179; <**0.001**
Normal LDH (*n* = 556)	53.9 (43.9–65.9)	67.1 (38.7–81.6)	105.0 (83.9–125.6)	0.322; <**0.001**
High LDH (*n* = 115)	26.8 (18.6–46.4)	NR (7.3-NR)	106.1 (71.7-NR)	0.295; <**0.001**
Low PC proliferative rate (*n* = 405)	47.8 (41.6–67.4)	72.3 (38.7-NR)	103.6 (80.4–130.6)	0.144; <**0.001**
High PC proliferative rate (*n* = 101)	32.9 (15.2–54.9)	21.5 (7.6–28.0)	62.5 (42.7–85.9)	0.217; **0.008**
PI-containing induction (*n* = 557)	54.3 (40.7–62.6)	NR (54.0-NR)	124.4 (79.9-NR)	**0.007**; <**0.001**
Others (*n* = 371)	45.2 (36.5–58.3)	67.1 (27.5–85.4)	106.1 (84.8–125.6)	0.405; <**0.001**
Early SCT (*n* = 466)	54.9 (45.3–66.5)	85.4 (72.3-NR)	130.6 (106.1-NR)	**0.038**; <**0.001**
Delayed or no SCT (*n* = 150)	77.2 (44.6-NR)	NR (38.7-NR)	125.6 (105.6-NR)	0.289; **0.001**

ISS indicates international staging system

*LDH* lactate dehydrogenase, *NR* not reached, *PC* plasma cell, *PI* proteasome inhibitor, and *SCT* stem cell transplant

^a^*P*-value for log-rank test in Kaplan-Meier analysis. The first value represents comparison between del(17p) and high-risk translocation groups and the second value represents comparison between del(17p) and standard-risk groups

The values given in bold represent *P*-values <0.05, which are considered statistically significant

### Impact of percentage of plasma cells with del(17p)

Data on percentage of PCs with del(17p) on FISH were retrievable in 260 patients with del(17p). The median PC% with del(17p) was 69.5 (range, 8–100). Taking a 60% cut-point, the median OS in those with del(17p) in ≥60% PCs (*n* = 153) vs. those with del(17p) in <60% clonal PCs (*n* = 107) were 36.8 months (95% CI, 30.9–51.4) vs. 54.0 months (95% CI, 42.0–65.9) (*P* = 0.432). The corresponding figures for median PFS were 15.1 months (95% CI, 10.4–17.9) and 25.7 months (95% CI, 22.1–31.9) (*P* = 0.009). The median PFS and OS for 20, 30, 40, and 50% cut-points are given in Supplementary material.

### Predictors of outcome in patients with de novo del(17p)

We performed univariable analysis with age ≥65 vs. <65 years, serum creatinine >2 vs. ≤2 mg/dL, bone marrow PC percentage ≥50 vs. <50%, ISS III vs. I/II stage, elevated vs. normal LDH, presence vs. absence of a HRT, presence vs. absence of monosomy 13, presence vs. absence of hyperdiploidy, high vs. low PC proliferation rate, PI-containing vs. other induction therapy and diagnosis of MM up to 2012 vs. later (there was a significant difference in OS using this cut-off; details given in Supplementary material) as independent variables to determine their association with PFS and OS. Variables with a *p*-value <0.1 in univariable analysis were included as potential predictors in multivariable Cox proportional hazards model and we arrived at a final model using stepwise backward elimination. To assess the impact of percentage of PCs with del(17p), we included each cut-point (viz. 20, 30, 40, 50, and 60%) with the above predictors. The results of the analysis are shown in Table 4. ISS stage III disease, elevated LDH and coexistent HRTs were associated with reduced OS, while percentage of PCs with del(17p) was not a significant predictor in the multivariable model. We stratified patients into three groups similar to the revised ISS: low-risk with ISS I stage, normal LDH, and no HRT (*n* = 34; 15.4%); intermediate-risk: neither low nor high-risk (*n* = 140; 63.3%); and high-risk: ISS III stage and either elevated LDH or coexistent HRT (*n* = 47, 21.3%)^6^. The low, intermediate and high-risk sub-groups within the del(17p) group had a median OS of 96.2 months (95% CI, 62.8-NR), 45.4 months (95% CI, 40.7–58.3) and 22.8 months (95% CI, 17.4–34.4) respectively (*P* < 0.001). The median PFS were 31.9 months (95% CI, 25.7–57.3), 22.3 months (95% CI, 18.4–28.3), and 9.8 months (95% CI, 5.5–15.5), respectively (*P* < 0.001) (Supplementary material).

**Table 4:** Effect of baseline characteristics on survival measures in patients with de novo del(17p) (*n* = 310)

Independent variable	Progression-free survival (PFS)			Overall survival (OS)		
	*p*-value for univariable analysis	HR (95% CI) for multivariable analysis	*p-*value for multivariable analysis	*p*-value for univariable analysis	HR (95% CI) for multivariable analysis	*p*-value for multivariable analysis
Age ≥65 vs. <65 years (147 vs. 163)	0.714	NI	–	0.010	1.40 (0.96–2.04)	0.079
Creatinine >2 vs. ≤2 mg/dL (52 vs. 231)	0.027	1.18 (0.66–2.00)	0.561	0.072	0.86 (0.49–1.47)	0.600
Bone marrow PCs ≥50% vs. <50% (164 vs. 140)	0.110	NI	–	0.536	NI	–
ISS III vs. I/II stage (93 vs. 154)	<0.001	1.89 (1.31–2.71)	**<0.001**	<0.001	2.08 (1.42–3.04)	**<0.001**
Elevated vs. normal LDH (49 vs. 157)	0.001	1.72 (1.13–2.53)	**0.012**	0.003	1.83 (1.16–2.80)	**0.009**
High-risk translocation vs. no high-risk translocation (75 vs. 235)	0.001	1.61 (1.10–2.32)	**0.015**	<0.001	1.53 (1.01–2.27)	**0.044**
Monosomy 13 vs. no monosomy 13 (163 vs. 147)	0.020	1.08 (0.75–1.57)	0.672	0.024	1.04 (0.63–1.72)	0.885
HRD vs. no HRD (112 vs. 198)	<0.001	0.88 (0.54–1.42)	0.614	0.038	0.79 (0.52–1.20)	0.277
High PC proliferative rate vs. low proliferative rate (42 vs. 98)	<0.001	1.56 (0.93–2.60)	0.090	0.042	0.92 (0.52–1.63)	0.788
PI-containing induction vs other induction therapy (219 vs. 89)	0.935	NI	–	0.472	NI	–
Diagnosis upto 2012 vs. later (173 vs. 137)	<0.001	0.52 (0.36–0.75)	**<0.001**	0.011	0.76 (0.47–1.19)	0.236
*Percentage of PCs with del(17p)*						
≥20% vs. <20% (224 vs. 36)	0.074	1.85 (0.96–4.13)	0.068	<0.001	1.07 (0.38–4.46)	0.916
≥30% vs. <30% (207 vs. 53)	0.057	1.91 (1.12–3.50)	**0.015**	<0.001	1.36 (0.65–3.34)	0.441
≥40% vs. <40% (193 vs. 67)	0.012	1.85 (1.15–3.12)	**0.009**	0.001	1.17 (0.60–2.49)	0.662
≥50% vs. <50% (170 vs. 90)	0.025	1.45 (0.96–2.26)	0.079	0.011	1.09 (0.62–2.03)	0.769
≥60% vs. <60% (153 vs. 107)	0.050	1.28 (0.89–1.88)	0.184	<0.001	1.18 (0.67–2.11)	0.570

The final multivariable model included 174 patients for PFS and 191 patients for OS for whom the parameters were available

Hyperdiploidy was defined as presence of trisomy involving ≥2 odd numbered chromosomes; the results were similar when presence of any trisomy/tetrasomy was used instead of HRD

*HR* hazard ratio, *HRD* hyperdiploidy, *ISS* international staging system, *LDH* lactate dehydrogenase, *NI* not included in analysis, *PC* plasma cell, and *PI* proteasome inhibitor.

The values given in bold represent *P*-values <0.05, which are considered statistically significant

## Discussion

We describe the outcomes of 310 MM patients with del(17p) treated at our center. Most patients received a PI-containing induction and more than half underwent a SCT. Seventy-six percent of patients attained a PR or better following induction, but the response rates were lower than those patients with HRT and SR disease. The median PFS and OS in the del(17p) group were 21and 47 months, respectively. The OS was dependent on the ISS stage and LDH level at diagnosis and presence of concurrent HRTs.

Patients with del(17p) had lower hemoglobin, higher PC proliferative rate, and higher LDH at diagnosis. This is consistent with prior observations from smaller datasets^8^. Compared to a cohort of 110 patients with del(17p) using 10% as the cut-off to define the abnormality, our cohort contained more patients above the age of 65 years and the proportion of patients with elevated LDH was lower (vs. 33%) in our cohort^30^. However, the percentage of patients with ISS III disease in our cohort were similar to the above study and another cohort of 110 patients where 60% was used as the cut-off to define presence of del(17p) (40–45%)^29,30^. The common cytogenetic abnormalities occurring in patients with del(17p) were abnormalities of chromosome 13, trisomies and t(11;14), and t(4;14) as reported previously^30^.

Most patients with del(17p) and HRT in our series received induction with a PI-based regimen, as bortezomib-based treatment has shown improvement in outcomes in patients with high-risk cytogenetics^47–49^. However, del(17p) patients were more likely to receive a PI + IMiD-based induction when compared to HRT-patients (39 vs. 24%). However, PI-based induction was not a predictor for improved PFS or OS in patients with del(17p) in our analysis. Among patients who underwent SCT, patients with del(17p) were more likely to do so within the first year of starting treatment. An early SCT did not improve OS in transplant eligible patients with del(17p). However, we did not include SCT as a time dependent co-variate in our multivariable analysis for factors impacting OS in patients with del (17p). The OS in MM has improved consistently over several years^2^. In this study, the OS in patients with del(17p) MM diagnosed in 2013 or later did not show improvement compared to those diagnosed before 2013, when controlled for other prognostic variables. Patients with del(17p) MM represent a cohort with unmet needs in the current era and our results call for better understanding of disease biology and development of new therapeutic strategies.

The inferior PFS and OS in patients with del(17p) observed in our cohort is consistent with previous observations^7–9,11,14,15,19,20,24–31^. The PFS was similar in patients with del(17p) and HRT, suggesting that the effect of initial therapy might be similar in both the subgroups. However, the median OS in patients with del(17p) was inferior suggesting that patients with del(17p) are at higher risk of relapse and developing treatment refractory disease. Concurrent genetic abnormalities such as high-risk translocations might be contributing to this effect, as shown by loss of significance for difference in OS when patients with del(17p) and no HRT are compared to patients with HRT, and the inferior OS in patients with concurrent del(17p) and HRT when compared to the above two groups. This further supports the cumulative nature of adverse impact resulting from high-risk cytogenetics observed in previous studies^28,29,31,41^. Also, our series contained 21 patients with relative loss of 17p, whose prognosis is not clearly defined in the literature. We show that their PFS and OS are similar to the general del(17p) group. So they should be considered to have high-risk FISH when making treatment decisions.

Finally, we show that patients with del(17p) are heterogeneous with regard to OS. The revised ISS system categorizes patients with del(17p) into either stage II or III^6^. In multivariable analysis, ISS stage, LDH, and concurrent high-risk translocations at diagnosis were associated with OS, and can be used to separate an (ultra) high-risk group (~21%) with ISS III stage and either high LDH or high-risk translocations with a median OS less than 2 years (estimated 5-year OS of 10% as against 40% for RISS stage III MM) who are candidates for aggressive therapy. The data also raises the question as to whether patients with concurrent HRT and del(17p) abnormality (~26% 5-year OS) should be in the ISS stage 3 group, irrespective of their LDH or ISS stage. Patients with ISS I stage, normal LDH and no high-risk translocation enjoy a median OS of 8 years, suggesting that close monitoring of patients with smoldering MM and monoclonal gammopathy of undetermined significance with del(17p) and early initiation of therapy might be beneficial^50–52^. ISS III has been previously identified as an adverse prognostic factor in patients with del(17p) while gain 19q13 was associated with improved survival^30^. Del(1p) and more than eight numerical aberrations detected, both by single nucleotide polymorphism array, exerted negative and positive impact on OS in patients with del(17p)^29^. However, the impact of high-risk translocations has not been demonstrated before in patients with del(17p).

We used the cut-offs used in our diagnostic laboratory based on the background positivity, to define del(17p). Different groups have suggested different cut-offs ranging from 10 to 60% to define del(17p) and the best cut-off is contentious^9,15,49,53^. Our study shows that when considered together with other adverse risk factors, the size of the PC clone with del(17p) has no impact on OS. Previous studies which defined optimal cut-offs for del(17p) did not consider the impact of coexistent high-risk factors^9^. We suggest that all patients with del(17p) as defined by a PC percentage with del(17p) higher than the background detection rate for FISH for the laboratory, should be treated as high-risk MM irrespective of the clone size.

Our study has limitations associated with a retrospective design such as missing data on baseline characteristics. Patients in all the three groups were not treated with similar therapy. But, a similar proportion of patients in the del(17p) and HRT groups received PI-based induction and upfront SCT, making these two groups similar with respect to treatment. We did not have data for 1q gain and del(1p) for patients before 2014 and so we did not include them in the prognostic models. Also, we did not include SCT and maintenance therapy as a time dependent covariates in the statistical models. We included patients with t(14;20) in the HRT-group. However, data for t(14;20) are controversial as it is a rare abnormality. The revised ISS does not consider such patients high-risk^6^. At our institution, we regard this as a high-risk abnormality^5^.

In conclusion, our series confirms the poor prognosis associated with del(17p). Patients with relative loss of 17p also carry a poor prognosis. We show that additional high-risk translocations, and high tumor burden modify the outcomes in patients with MM with del(17p). We suggest that patients with del(17p) and HRT be reclassified under stage III in revised-ISS. Dedicated clinical trials aimed at achieving minimal residual disease negativity are required to identify optimal therapeutic strategies in MM patients with del(17p)^54,55^.

## Supplementary information

Supplemental material
