# Supplementary material for: Natural history of multiple myeloma with de novo del(17p)
Source: Blood Cancer J. 2019 Mar 7;9(3):32. doi: 10.1038/s41408-019-0191-y (PMC6405846; doi:10.1038/s41408-019-0191-y)
Supplement: Supplementary file 1 — Supplemental material [file 41408_2019_191_MOESM1_ESM.pdf]

SUPPLEMENTARY MATERIAL

for

Natural History of Multiple Myeloma with de novo del(17p)

**Supplementary Table 1** - Induction regimens used in the study patients			
	De novo del(17p)	High-risk translocations	Standard-risk
PI-based	219 (71.1)	58 (73.4)	280 (51.8)
• PI+IMiD	119 (38.6)	19 (24.0)	103 (19.0)
• PI	100 (32.5)	39 (49.4)	177 (32.8)
IMiD-based	79 (25.6)	20 (25.3)	248 (45.8)
Others	10 (3.2)	1 (1.3)	13 (2.4)
P<0.001 for Fisher's exact test; IMiD indicates immunomodulatory drug; and PI, proteasome inhibitor.			

**Supplementary Table 2 - Best response to induction therapy (n=902)**				
Best response to therapy	De novo del(17p) (n=289)	High-risk translocations (n=79)	Standard-risk (n=534)	*P**
CR+VGPR	118 (40.8)	46 (58.2)	218 (40.8)	0.012
CR+VGPR+PR (ORR)	221 (76.5)	69 (87.3)	453 (84.8)	0.006
CR+VGPR+PR+MR (CBR)	247 (85.5)	76 (96.2)	495 (92.7)	<0.001
sCR	14 (4.8)	9 (11.4)	29 (5.4)	
CR	9 (3.1)	5 (6.3)	28 (5.2)	
VGPR	95 (32.9)	32 (40.5)	162 (30.3)	
PR	103 (35.6)	23 (29.1)	234 (43.8)	
MR	26 (9.0)	7 (8.9)	42 (7.9)	
SD	35 (12.1)	3 (3.8)	34 (6.4)	
PD	7 (2.4)	0 (0)	5 (1.0)	
CBR indicates clinical benefit rate; CR, complete response; MR, minimal response; ORR, overall response rate; PD, progressive disease; PR, partial response; sCR, stringent CR; SD, stable disease; and VGPR, very good partial response.*P for Fisher's exact test.				

Supplementary figure 1- **Survival outcomes in patients with del(17p13.1) and monosomy 17.** Kaplan Meier curves showing comparison of (a) Progression-free survival (PFS) and (b) overall survival (OS) between patients with del(17p13.1) (n=246) and monosomy 17 (n=41). Two patients with concurrent del(17p13.1) and monosomy 17 were excluded. For PFS, P= 0.486 for del(17p13.1) vs. monosomy 17; for OS, P=0.832 for del(17p13.1) vs. monosomy 17.

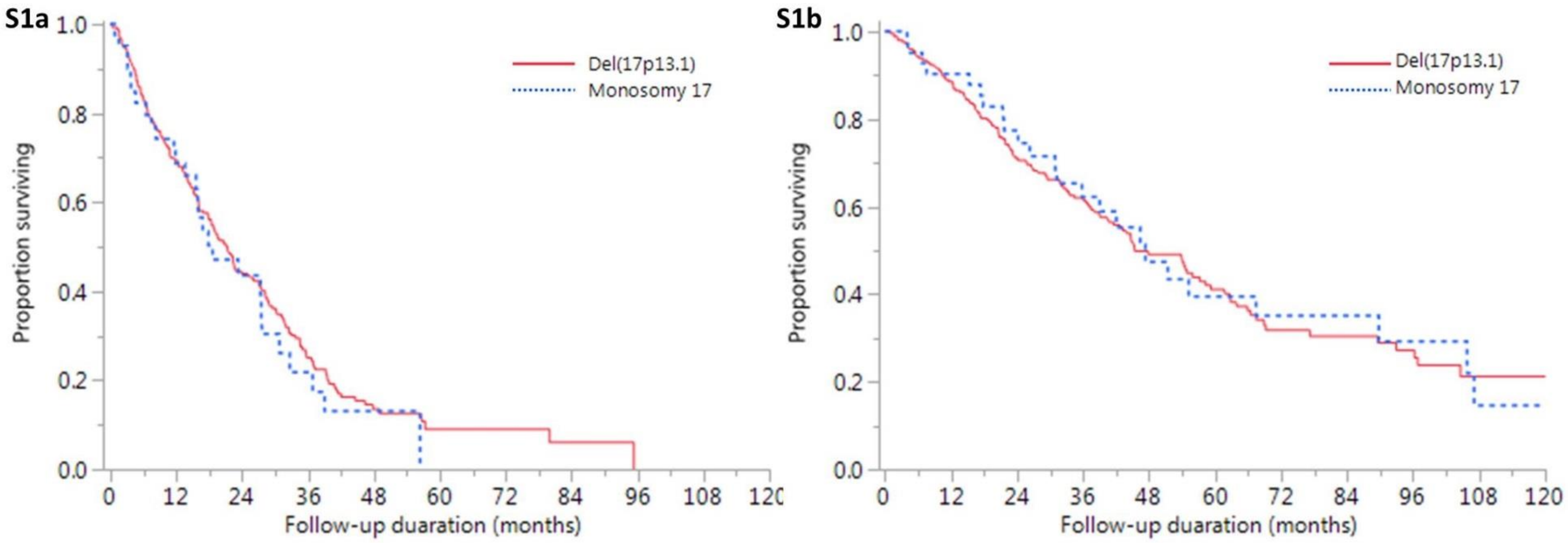

Del(17p)	246	147	85	40	14	5	4	2	0	0	0	246	213	156	114	68	44	26	22	16	5	1
Monosomy 17	41	25	10	5	1	0	0	0	0	0	0	41	37	27	20	12	9	7	6	5	2	1

Supplementary figure 2- **Survival outcomes in patients with del(17p13.1) and/or monosomy 17 [del(17p)] and relative loss of 17p.**

Kaplan Meier curves showing comparison of (a) Progression-free survival (PFS) and (b) overall survival (OS) between patients with del(17p) (n=289) and relative loss of 17p (n=21). For PFS, P= 0.485 for del(17p) vs. relative loss of 17p; for OS, P=0.603 for del(17p) vs. relative loss of 17p.

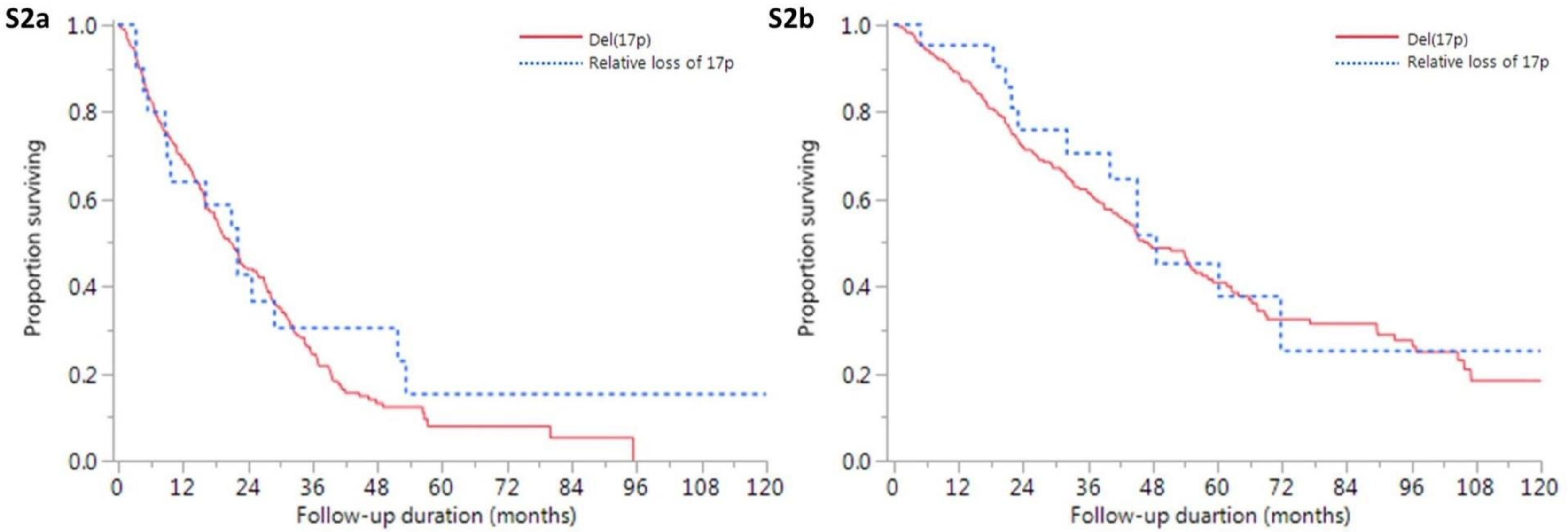

	0	12	24	36	48	60	72	84	96	108	120
Del(17p)	289	173	96	45	15	5	4	2	0	0	0
Relative loss of 17p	21	12	7	4	4	1	1	1	1	1	1

	0	12	24	36	48	60	72	84	96	108	120
Del(17p)	289	252	185	135	80	53	33	28	21	7	2
Relative loss of 17p	21	20	15	12	8	6	2	2	2	1	1

Supplementary figure 3- Survival outcomes in patients with del(17p) and high-risk translocation. Kaplan Meier curves showing comparison of (a) Progression-free survival (PFS) and (b) overall survival (OS) between patients with del(17p) and no high-risk chromosomal translocation (n=235), high-risk translocation (HRT) (n=79) and del(17p) with high-risk translocations (n=75). The median PFS in the three groups were 22.9 months (95% CI, 20.6-27.5), 22.0 months (95% CI, 16.7-26.8) and 14.7 months (95% CI, 9.8-17.9) respectively (P= 0.951 for del(17p) with no high-risk translocation vs. HRT; P<0.001 for del(17p) with no high-risk translocation vs. del(17p) with high-risk translocation). The median OS in the three groups were 54.9 months (95% CI, 45.4-64.0), 79.1 months (95% CI, 53.9-NR) and 29.5 months (95% CI, 20.0-38.1) respectively (P=0.052 for del(17p) with no high-risk translocation vs. HRT; P<0.001 for del(17p) with no high-risk translocation vs. del(17p) with high-risk translocation)

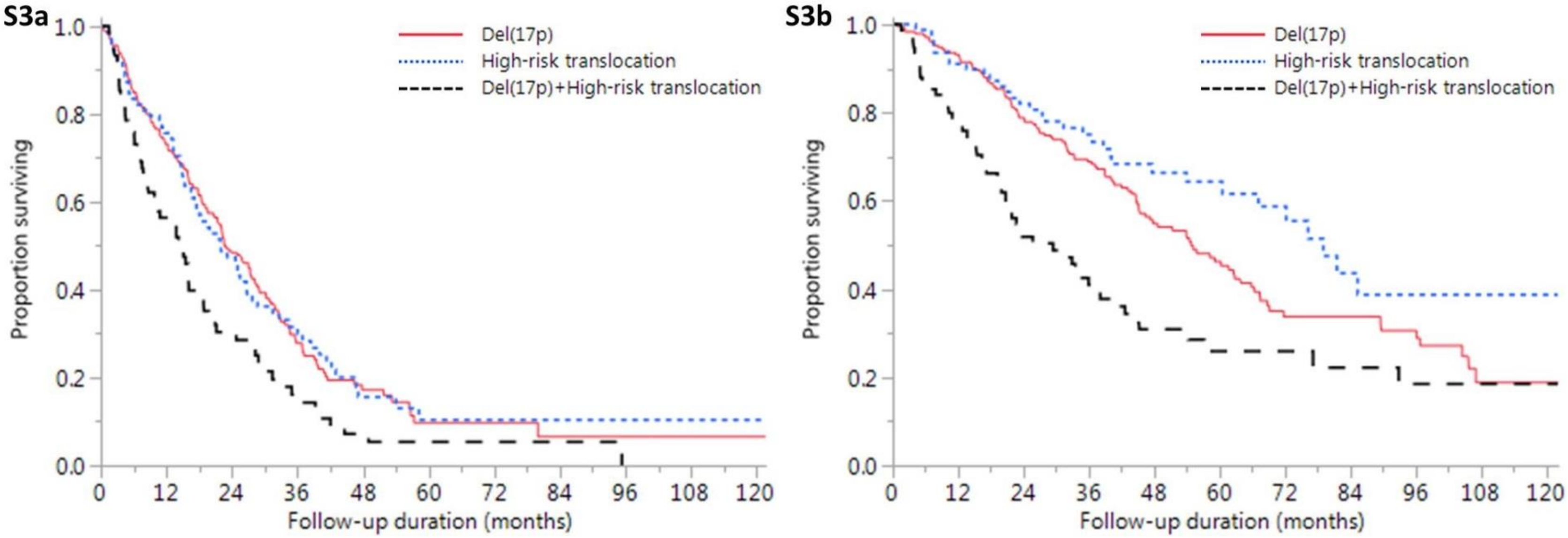

	0	12	24	36	48	60	72	84	96	108	120
Del(17p)	235	147	85	40	15	5	4	2	1	1	1
HRT	79	58	34	19	6	4	4	2	1	1	1
Del(17p)+HRT	75	38	18	9	4	1	1	1	0	0	0

	0	12	24	36	48	60	72	84	96	108	120
Del(17p)	235	215	164	121	70	49	27	24	18	6	2
HRT	79	71	62	48	33	24	18	9	6	5	4
Del(17p)+HRT	75	57	36	26	18	10	8	6	5	2	1

Supplementary figure 4- **Survival outcomes in patients with del(17p) based on year of diagnosis.** Kaplan Meier curves showing comparison of overall survival (OS) between patients categorized according to the year of diagnosis of multiple myeloma. (a)Comparison of patients diagnosed during 2004-2007, 2008-2012 and 2013-2016. *P-value for log rank test across the three groups and **P-value for log rank test between 2004-2007 and 2008-2012. The difference in OS across groups is driven by the improved survival in the 2013-2016 group. (b) Comparison of patients diagnosed between 2013-2016 and 2004-2012 showing significant difference in OS.

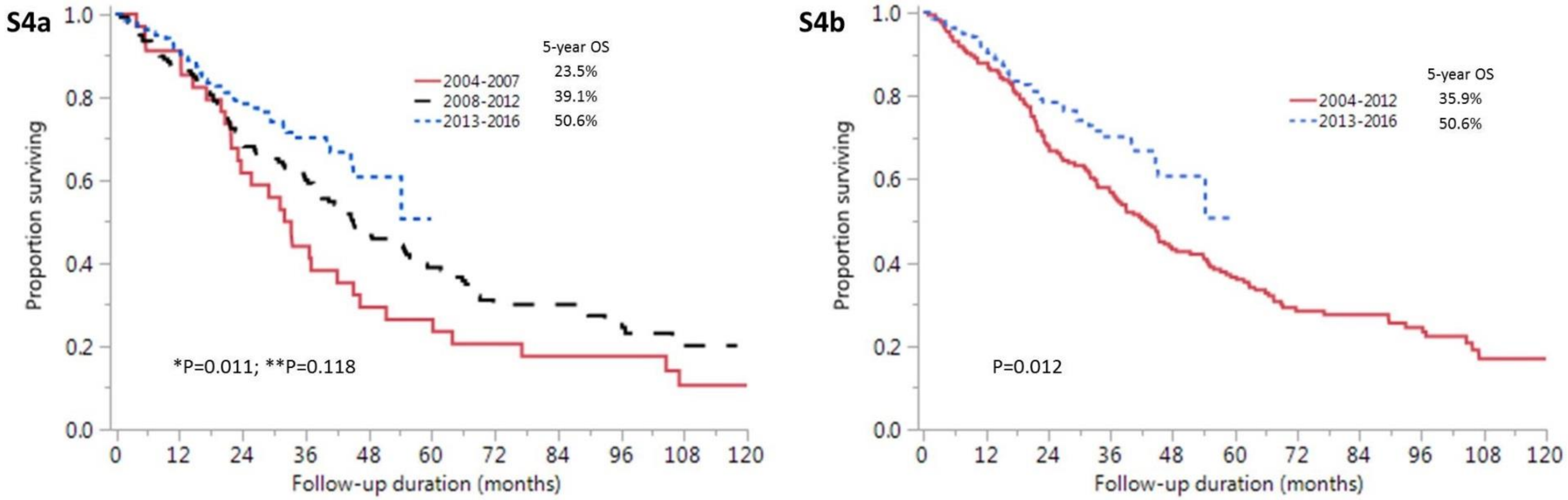

2004-2007	34	31	21	15	10	8	7	5	5	3	3
2008-2012	139	120	95	81	63	50	28	25	17	5	1
2013-2016	137	121	84	51	15	1	-	-	-	-	-

2004-2012	173	151	116	96	73	59	35	30	23	8	3
2013-2016	137	121	84	51	15	1	-	-	-	-	-

Supplementary figure 5- Survival outcomes in patients with del(17p) based on ISS, LDH and concurrent high-risk translocation. Kaplan Meier curves showing comparison of (a) Progression-free survival (PFS) and (b) overall survival (OS) between patients categorized into low-risk (ISS I stage, normal LDH and no high-risk translocation; n=34); intermediate-risk (neither low nor high-risk; n=140); and high-risk (ISS III stage and either elevated LDH or coexistent high-risk translocation; n=47).

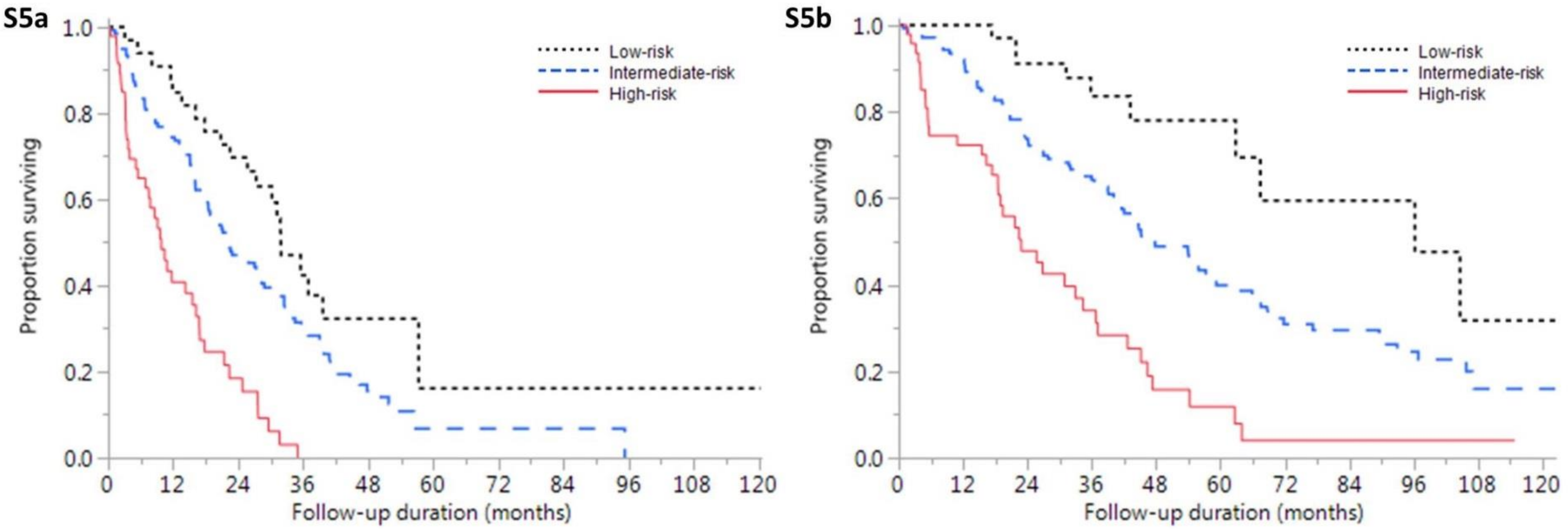

S5a:

Low-risk	34	28	22	9	4	2	1	1	1	1	1
Intermediate-risk	140	91	51	30	11	2	2	2	0	0	0
High-risk	47	16	6	0	0	0	0	0	0	0	0

S5b:

Low-risk	34	34	31	20	11	9	6	6	5	1	1
Intermediate-risk	140	127	98	79	50	33	23	19	14	4	2
High-risk	47	33	18	12	5	3	1	1	1	1	0

Supplementary Table 3- Progression free survival and overall survival for patients stratified by cut-point for del (17p) (n=260)				
	PFS	***P****	***OS***	***P****
• ≥20% vs. <20% (224 vs. 36)	19.2 (16.1-22.9) vs. 32.5 (21.1-36.6)	0.087	45.3 (40.5-55.2) vs. NR (43.9-NR)	**0.002**
• ≥30% vs. <30% (207 vs. 53)	18.8 (16.1-22.6) vs. 30.8 (21.6-36.6)	0.065	45.2 (38.1-54.6) vs. 89.6 (53.9-NR)	**0.001**
• ≥40% vs. <40% (193 vs. 67)	18.3 (15.9-22.1) vs. 30.8 (22.3-33.7)	**0.014**	45.2 (37.1-54.6) vs. 89.6 (43.9-NR)	**0.002**
• ≥50% vs. <50% (170 vs. 90)	17.8 (15.1-21.0) vs. 30.3 (22.3-32.5)	**0.027**	44.8 (35.8-55.2) vs. 58.3 (45.2-96.2)	**0.013**
• ≥60% vs. <60% (153 vs. 107)	16.8 (14.5-19.2) vs. 28.3 (22.5-31.8)	0.051	38.1 (31.9-54.3) vs. 58.3 (47.3-96.2)	**<0.001**
*P for log rank test; NR, not reached.				
